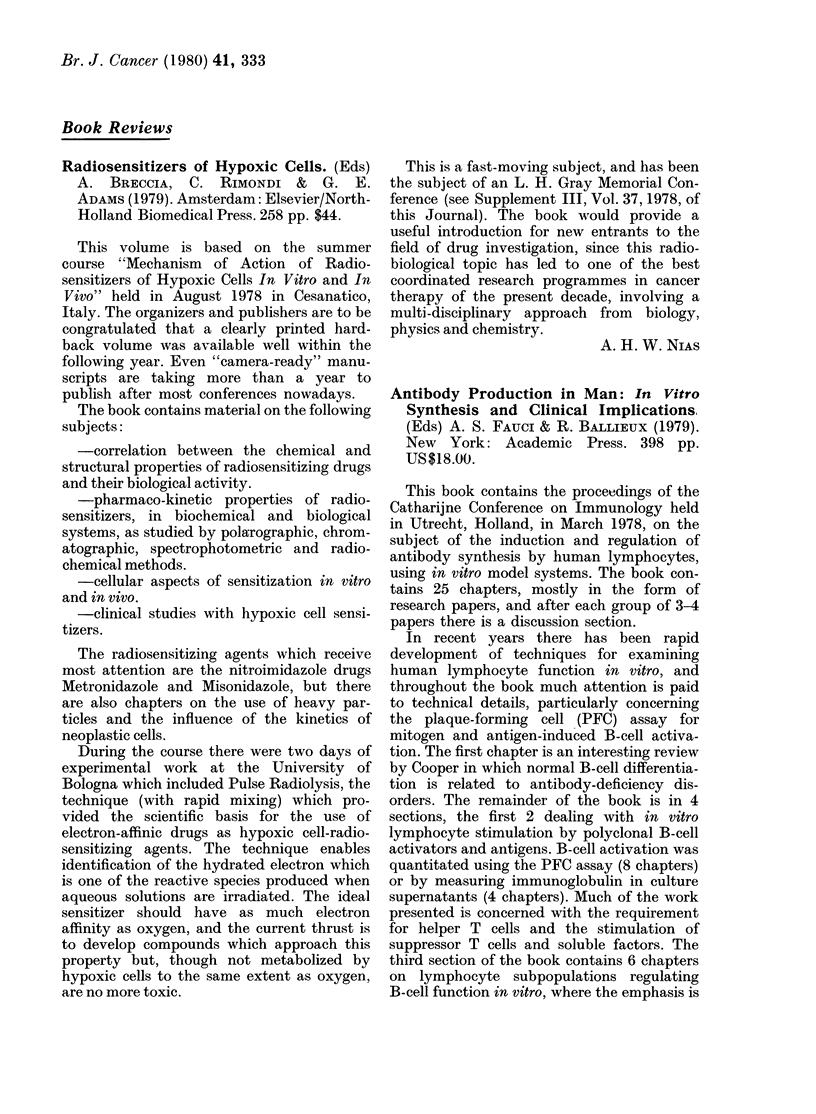# Radiosensitizers of Hypoxic Cells

**Published:** 1980-02

**Authors:** A. H. W. Nias


*Br. J. Cancer* (1980) **41**, 333

### *Book Reviews*

**Radiosensitizers of Hypoxic Cells.** (Eds) A. BRECCIA, C. RIMONDI & G. E. ADAMS (1979). Amsterdam: Elsevier/North-Holland Biomedical Press. 258 pp. $44.

This volume is based on the summer course "Mechanism of Action of Radiosensitizers of Hypoxic Cells *In Vitro* and *In Vivo*" held in August 1978 in Cesanatico, Italy. The organizers and publishers are to be congratulated that a clearly printed hard-back volume was available well within the following year. Even "camera-ready" manuscripts are taking more than a year to publish after most conferences nowadays.

The book contains material on the following subjects:

—correlation between the chemical and structural properties of radiosensitizing drugs and their biological activity.

—pharmaco-kinetic properties of radiosensitizers, in biochemical and biological systems, as studied by polarographic, chromatographic, spectrophotometric and radiochemical methods.

—cellular aspects of sensitization *in vitro* and *in vivo*.

—clinical studies with hypoxic cell sensitizers.

The radiosensitizing agents which receive most attention are the nitroimidazole drugs Metronidazole and Misonidazole, but there are also chapters on the use of heavy particles and the influence of the kinetics of neoplastic cells.

During the course there were two days of experimental work at the University of Bologna which included Pulse Radiolysis, the technique (with rapid mixing) which provided the scientific basis for the use of electron-affinic drugs as hypoxic cell-radiosensitizing agents. The technique enables identification of the hydrated electron which is one of the reactive species produced when aqueous solutions are irradiated. The ideal sensitizer should have as much electron affinity as oxygen, and the current thrust is to develop compounds which approach this property but, though not metabolized by hypoxic cells to the same extent as oxygen, are no more toxic.

This is a fast-moving subject, and has been the subject of an L. H. Gray Memorial Conference (see Supplement III, Vol. 37, 1978, of this Journal). The book would provide a useful introduction for new entrants to the field of drug investigation, since this radiobiological topic has led to one of the best coordinated research programmes in cancer therapy of the present decade, involving a multi-disciplinary approach from biology, physics and chemistry.

A. H. W. NIAS